# Polysomnographically Defined Restless Sleep Disorder and Periodic Limb Movements during Sleep in Children Born Prematurely

**DOI:** 10.3390/children11060658

**Published:** 2024-05-28

**Authors:** Lourdes M. DelRosso, Hovig Artinian, Maria P. Mogavero, Oliviero Bruni, Manisha Witmans, Mary Anne Tablizo, Michelle Sobremonte-King, Raffaele Ferri

**Affiliations:** 1 University of California San Francisco, 155 N. Fresno St, Fresno, CA 93701, USAmtablizo@stanford.edu (M.A.T.); 2 Seattle Childrens Hospital, Seattle, WA 98105, USA; michelle.sobremonte-king@seattlechildrens.org; 3 Sleep Disorders Center, Division of Neuroscience, San Raffaele Scientific Institute, 20127 Milan, Italy; paola_mogavero@libero.it; 4 Vita-Salute San Raffaele University, 20132 Milan, Italy; 5 Department of Developmental and Social Psychology, Sapienza University, 00185 Rome, Italy; oliviero.bruni@uniroma1.it; 6 Department of Pediatrics, University of Alberta, Edmonton, AB T6G 1C9, Canada; mwitmans@ualberta.ca; 7 Department of Pediatrics, Division of Pediatric Pulmonary and Sleep Medicine, Stanford University, Palo Alto, CA 94305, USA; 8 Sleep Research Centre, Department of Neurology IC, Oasi Research Institute-IRCCS, 94018 Troina, Italy; rferri@oasi.en.it

**Keywords:** restless sleep disorder, periodic limb movement during sleep, prematurity

## Abstract

Introduction: Children born prematurely (<37 weeks’ gestation) are at increased risk of perinatal complications, comorbidities, and iron deficiency. Iron deficiency is associated with restless legs syndrome and periodic limb movement disorder. In this study, we assessed the prevalence of restless sleep disorder (RSD) and elevated periodic limb movements during sleep (PLMS) in children born prematurely who underwent polysomnography. Methods: A retrospective chart review of sleep studies was conducted in children aged 1–18 years (median age 4 years) with a history of premature birth. Children with genetic syndrome, airway surgery, or tracheostomy were excluded. Three groups were compared: children with PLMS index >5, children with RSD, and children with neither elevated PLMS index nor RSD. Results: During the study, 2577 sleep studies were reviewed. Ninety-two studies fit our criteria and were included in the analysis. The median age at birth was 31 weeks, and the interquartile range (IQR) was 27–34 weeks. A total of 32 (34.8%) children were referred for restless sleep and 55 (59.8%) for snoring. After polysomnography, 18% were found to have a PLMS index >5/h, and 14% fit the criteria for restless sleep disorder (RSD). There were no statistically significant differences in PSG parameters among the children with RSD, PLMS, and the remaining group, except for lower obstructive apnea/hypopnea index (Kruskal–Wallis ANOVA 8.621, *p* = 0.0135) in the RSD group (median 0.7, IQR 0.3–0.9) than in the PLMS (median 1.7, IQR 0.7–3.5) or the non-RSD/non-PLMS (median 2.0, IQR 0.8–4.5) groups. Conclusions: There was an elevated frequency of RSD and elevated PLMS in our cohort of children born prematurely. Children born prematurely are at higher risk of iron deficiency which can be a contributor factor to sleep -related movement disorders. These results add new knowledge regarding the prevalence of RSD and PLMS in these children.

## 1. Introduction

The World Health Organization defines preterm birth or prematurity when birth occurs earlier than 37 weeks gestational age or less than 259 days [1]. The reported prevalence of premature birth is approximately 5 to 18% of births. Further classifications based on gestational age include extremely preterm (birth < 28 weeks), very preterm (birth between >28 and 32 weeks), moderately preterm (birth at >32 to 33 weeks), and late preterm (34–36 weeks).

Preterm birth or prematurity is associated with a higher risk of complications during birth and postnatal comorbidities, including pulmonary consequences due to alveoli underdevelopment, such as bronchopulmonary dysplasia or chronic lung disease, cerebral palsy, and seizures, among others. Despite improvements in the management of perinatal complications, the trajectory of the long-term consequences of prematurity is still under investigation [2]. Studies have shown that adults with a history of prematurity are more vulnerable to kidney disease, obstructive lung disease including asthma, neurodevelopmental disorders, and endocrine disorders [3]. While neonatal mortality and morbidity have improved in infants born prematurely, there has only been a slight improvement in the incidence of neurodevelopmental disability [4]. Male infants and those born extremely premature are at the highest risk of adverse neurodevelopmental outcomes [5]. Children born prematurely also demonstrate decreased gray matter, white matter, basal ganglia, and cerebellar volumes; to the extent that data are available, infants born prematurely exhibit differences in electroencephalographic spectral values, total sleep time, and arousal thresholds as compared to infants born at full term [6].

Preterm infants are at higher risk of iron deficiency due to their inadequate iron storage resulting from preterm birth, early onset of postnatal erythropoiesis, and rapid growth after birth [7]. Preterm infants are at higher risk of dysregulation of processes related to iron, which is implicated in movement disorders [7]. Iron is a crucial nutrient that contributes to fetal and neonatal brain development and is associated with critical cellular processes in the immature brain, including the maintenance of neural cell energy status, myelination, and monoamine neurotransmitter homeostasis [7]. There is a highly complex relationship between iron acquisition and myelin production. The higher metabolic state and increased oxygen consumption in neonates are also suggested as being iron-dependent. These disruptions in processing and the affected brain development could lead to permanent adverse consequences, which can contribute to an increased risk of movement disorders in children born prematurely. The prevalence of iron deficiency anemia is not known in premature infants but is commonly reported.

Only a few studies have looked at sleep consequences in children or adults born prematurely. In a cohort study comparing adults born prematurely versus full-term controls, the risk of chronic snoring was 2.2 times higher in the very-low-birth-weight premature group compared to the control group [8]. A large national cohort in Sweden also demonstrated an increased risk of sleep-disordered breathing in adults with a history of prematurity, with those born extremely premature having a higher adjusted hazard ratio of 2.63 (CI, 2.41–2.87) [9]. Our group previously reported an increased periodic limb movement index during sleep (PLMS index) in infants born prematurely [10].

In this study, we aimed to examine polysomnographic studies in older children with a history of prematurity to assess the presence and severity of sleep-related movement disorders. We hypothesized that older children with a history of prematurity might experience increased motor activity during sleep, as reflected by the PLMS index and large muscle group movement (LMM) index [11], which characterize the newly described restless sleep disorder (RSD) [12,13]. The knowledge obtained from this study contributes to the scientific body of evidence, adding prematurity as a potential contributor to an increased risk of sleep-related movement disorders in children.

## 2. Methods

### 2.1. Subjects

This was a retrospective chart review of sleep studies of children aged 1–18 years evaluated by a sleep clinician at the Seattle Children’s Hospital Sleep Center. Selection criteria included diagnostic sleep studies performed from July 2021 to July 2022, at Seattle Children’s Hospital, Seattle, WA, USA, in children with a history of prematurity documented in the chart. The exclusion criteria were children with genetic syndromes, studies other than diagnostic polysomnography (titration of positive airway pressure therapy, split night studies, studies on children on mechanical ventilation), children with tracheostomy, or oxygen-dependent children, and infants younger than 12 months. The local ethics committee approved the study.

### 2.2. Polysomnography

The PSG studies reviewed were performed at Seattle Children’s Hospital, Seattle, WA, USA, according to the American Academy of Sleep Medicine (AASM) criteria [13], scored by a certified sleep technologist, and interpreted by a board certified sleep physician. The data were recorded using a Sandman Elite Natus system XLTek Natus Polysomnography System, 2007, Ontario, Canada. Parameters recorded included electroencephalogram (EEG: two frontal, two central, and two occipital channels, referred to as the contralateral mastoid); electrooculogram, electromyogram (EMG) of the submentalis muscle, EMG right and left tibialis anterior muscles, respiratory signals such as oronasal thermistor and nasal pressure transducer, effort signals for thorax and abdomen by inductance plethysmography, arterial oxygen saturation with pulse waveforms, capnography, a single-lead electrocardiogram, video, and audio recording. Calibrations were performed per routine standards by sleep technicians. A certified sleep technologist scored sleep stages and PLMS, which were interpreted by a board-certified sleep physician according to the AASM criteria [14]. 

Limb movement was scored if there was an increase in EMG amplitude >8 µvolts above the resting EMG in either the right or left anterior tibialis and lasted 0.5–10 s. If limb movement occurred as part of a series >4, within 5 to 90 s between each movement, then it was scored as part of periodic limb movement. Abnormal periodic limb movement during sleep index (PLMI) is defined by ≥5/h in pediatric population [14]. 

The large muscle movement index (LMM) was calculated per current criteria as recommended by the International Restless Legs Syndrome Study Group (IRLSSG) executive committee [11]. The committee also formulated diagnostic criteria for the newly described pediatric entity, restless sleep disorder [12]. Large muscle movement was scored if there was a temporally overlapping increase in EMG activity and/or the occurrence of movement artifact in any combination of at least two recommended channels (tibialis anterior muscle EMG, chin EMG, EEG, EOG, ECG). The increase in EMG activity or movement artifact signal had to be at least twice the amplitude of the background signal amplitude and was scored after at least 10 s of sleep without movement. The minimum duration of an LMM is 3 s, and the maximum duration is 30 s for children and 45 s for adults. An LMM index of ≥5 is considered abnormal [11]. 

### 2.3. Statistical Analysis

Descriptive statistics were calculated using commercially available software STATISTICA v.6, StatSoft Inc., Tulsa, OK, USA. Because of the non-normal distribution of the data, nonparametric descriptives were used. The chi-square test was used to compare frequencies. To test the eventual effect of gestational age at birth on the PLMS index or LMM index, we also carried out two multiple regression analyses by considering these factors as dependent variables. A set of independent predictors that could influence these parameters, such as gestational age at birth, age at PSG, sleep efficiency, arousal index, oAHI, and LMM index if the dependent variable was the PLMS index, and vice versa, were also included. F correlation coefficients of 0.1 were considered small, 0.3 medium, and 0.5 or above large based on Cohen [15]. Statistical significance was set at *p* < 0.05. 

## 3. Results

During the study, 2577 total sleep studies were reviewed, and 162 were conducted in children with a documented history of prematurity (6%). The following were excluded: 25 sleep studies were split night studies or titration studies (14%), 29 sleep studies were from children with syndromes (Down, Prader–Willi, Wiedmann–Beckmann, achondroplasia), 4 studies were conducted on children with tracheostomy tube, 6 in infants aged <12 months, and 6 were post-airway interventions.

Ninety-two diagnostic studies were included in the analysis. The median gestational age at birth was 31 weeks, and the interquartile range (IQR) was 27–34 weeks.

A total of 32 (34.8%) children were referred for restless sleep and 55 (59.8%) for snoring; the remaining 5 were referred for insomnia. Sixteen (17.4%, 2 girls and 14 boys) had a PLMS index >5/h (median 8.7, interquartile range 6.6–13.5), 13 (14.1%, 6 girls and 7 boys) had an LMM index >5/h (median 6.2, interquartile range 5.5–7.5); the remaining 63 children, including 22 girls and 41 boys (sex distribution among groups: chi-square 4.18, NS) did not have RSD or an elevated PLMS. Altogether, the children with an LMM index >5/h and those with a PLMS index >5/h had restless sleep as an indication for their sleep study in 72.4% of cases (21 out of 29), while snoring was the prevalent indication in the non-RSD/non-PLMS group (48 out of 63, chi-square 76.2, *p* < 0.000001). The details on the demographics and gestational age at birth of the patients, subdivided by sex, are reported in Table 1.

Table 2 shows the descriptive statistics of the PSG parameters obtained in our group of children born prematurely.

The results of the multiple regression analysis between the PLMS index or LMM index (used as dependent variables) and the selected PSG parameters (used as independent factors) in children born prematurely are reported in Table 3. The PLMS index was positively correlated with gestational age at birth (with a small-to-medium correlation coefficient), although this did not reach statistical significance; however, it was negatively correlated with sleep efficiency (with a similar medium-size correlation coefficient, although not significant) and negatively correlated with oAHI (with a medium-to-large and significant correlation coefficient). Conversely, the LMM index did not appear to be correlated with gestational age at birth. However, it was negatively and significantly correlated with oAHI (again with a medium-to-large correlation coefficient) and positively (but not significantly) correlated with the arousal index and age (with medium-size correlation coefficients). There was also a negative correlation between the LMM and PLMS indices (with a small-to-medium correlation coefficient), although this correlation did not reach statistical significance.

## 4. Discussion

In this study, we found a high frequency of RSD (14.1%) and a PLMS index >5/h (17.4%) in children born prematurely. We previously reported that among children referred to the Seattle Children’s Hospital Sleep Center, the prevalence of RSD was 7.7% and that of PLMS is 9.3%. This finding is consistent with those of Cielo et al., who reported an increased prevalence of PLMD in children born prematurely compared to that in the general pediatric population. The same study also found a high prevalence of RLS in children born prematurely [16].

This increased risk of PLMS and RSD in children born prematurely could be secondary to the high risk of iron deficiency during infancy. Prematurity, maternal anemia, and low birth weight are known risk factors for anemia in infancy [17]. Several factors can contribute to iron deficiency in patients with a history of prematurity. Preterm infants have low iron stores at birth, mainly because most of the iron deposition occurs in the third trimester, and this would be curtailed for preterm infants. The iron metabolic demands with increased oxygen consumption increase substantially with rapid growth. Anemia can develop in some preterm babies due to frequent blood withdrawal for laboratory evaluations and increased risk of bleeding. Furthermore, the iron stores are quickly used up in the first eight weeks of life. Iron deficiency can also result from nutritional deficiency from poor absorption. It can also occur as a result of anemia owing to chronic disease, such as illness during the course management of bronchopulmonary dysplasia associated with infections, renal disease, and inflammatory conditions.

Iron is essential for many processes of brain development, and anemia during early neonatal life could have implications for some of the findings noted in this study. In animal models, iron deficiency is linked to deficits in the striatal dopamine system, demonstrating adverse development of the basal ganglia system, which plays critical roles in the initiation and control of movement, as well as in the hippocampus and cortex, which are crucial for the functions of memory and cognition [18]. The critical time for the need for iron for myelination and the development of critical neural pathways, which are iron-dependent, was reported by Wang et al. [7]. Preterm infants with anemia (Hb ≤ 10 g/dL) and low iron stores (serum ferritin ≤ 76 μg/L) were found to have an increased number of abnormal neurologic reflexes (such as glabella reflex, Babinski reflex, plantar grasp, palmar grasp, passive movement of the arms, and passive movement of the legs) at 37 weeks’ gestational age compared with nonanemic, iron-replete infants [19]. Specific motor deficits such as fidgety movements (the movements could occur continuously in awake infants except during fussing and crying and mainly refer to the small-amplitude, moderate-speed, and variable acceleration that occur in the neck, trunk, and limbs in all directions) seem to predominate in preterm infants compared to healthy-term infants [20].

We previously identified increased iron deficiency in infants born prematurely [10]. Studies in children with a history of iron deficiency during infancy reported the persistence of increased leg muscle activation and leg movements during sleep when measured at school age [21]. While most studies on motor activity in infants born prematurely have focused mainly on identifying the predictors of cerebral palsy or neurologic deficits [22], polysomnographic studies focusing on sleep-related movement disorders are scarce.

Our study also shows an interesting correlation between PLMS with sleep efficiency and LMM and the arousal index, similar to that recently reported in healthy adults [23]. A previous study showed that infants born to mothers with anemia during pregnancy had shorter nocturnal sleep duration than infants born without anemia. Furthermore, infants born to mothers who took iron supplementation during pregnancy slept longer than infants born to mothers who did not take iron supplementation during pregnancy [24].

Iron deficiency is a well-established cause of restless legs syndrome and periodic limb movement disorder, and is a suspected contributor to RSD due to its importance in brain dopamine production and neurotransmitter system development [25]. Iron supplementation with oral ferrous sulfate (3 mg/kg) is the recommended treatment for children with RLS, RSD, or PLMD with ferritin levels lower than 50 mg/L. Intravenous iron supplementation also showed promising results [26]. Interestingly, however, adults with a history of very preterm birth but without macroscopic perinatal brain injury demonstrated no significant differences in presynaptic dopamine synthesis capacity from controls [27]. This raises the question as to whether prematurity is affected not as much by the amount of iron or dopamine produced but by structural or functional connections that may contribute to the increased PLMS and increased LMM seen in children born prematurely. It is fascinating to note that studies on premature children have highlighted alterations in the basal ganglia (6), such as studies on RLS and PLMS in adults [28]. This reinforces that the mechanisms of altered connectivity and neuroplasticity may contribute to the disorder’s pathogenesis. The implications of iron deficiency may involve CNS development much more broadly.

In our study, PLMS correlated positively with gestational age. This may indicate that the pathways involved in PLMS may not develop appropriately in premature infants. Most cerebral pathways and networks are built in the second half of gestation; thalamocortical pathways develop between 20 and 32 weeks, callosal and long cortico-cortical pathways between 24 and 35 weeks, while the maturation of oligodendroglia and myelination occur during the third trimester [29]. A full-term infant brain rearranges cortical inhibition excitation fibers and forms many new synapses. Prematurity compromises these neural maturation processes, limiting sensory-driven brain connectivity and synaptic pruning, potentially leading to future impairment [30]. This may explain the lack of improvement in the neurodevelopmental delays reported in adults with a history of prematurity.

Polysomnographic studies in children born prematurely have mainly focused on obstructive sleep apnea. Jaleel et al. [31] reported an increased AHI in children with a history of prematurity, but full polysomnographic parameters, including the PLMS index and arousal index, were not included in the study. Manuel et al. [32] also studied children born prematurely referred to otolaryngology and found increased respiratory comorbidities, but polysomnography was not performed. Questionnaire-based studies, including the Brief Infant Sleep Questionnaire, revealed that two-year-old children born prematurely were more restless during sleep and reported more sleep difficulties and shorter sleep duration correlated with increased motor activity. Furthermore, other daytime symptoms included lower attention and increased negative emotions [33]. A literature review of children born prematurely also demonstrated that reduced sleep efficiency and poor sleep quality tended to persist throughout life [34]. Our study also shows an interesting correlation between PLMS with sleep efficiency and LMM and arousal index, similar to that recently reported in healthy adults [23].

Another observation in our study was the younger age of our patient population, despite reviewing all charts of children undergoing polysomnography. This may indicate a younger referred population or that movements during sleep decrease with maturation [35,36].

The limitations to our study include it being a single-center experience and the retrospective nature of data collection, the unknown contribution of other comorbidities to the results, and the unknown iron status currently or at birth for all participants. The prevalence or severity of neonatal iron deficiency in these premature infants was unknown, as was the prevalence of iron deficiency at this study’s time. Nevertheless, these findings are interesting in that they suggest an increased prevalence of limb movement disorders in children born prematurely. In addition, the retrospective observational nature of this study, which used a convenience sample, did not allow a preventive power analysis. However, a post hoc power analysis showed that in order to be able to obtain a statistical significance for a medium-size correlation of 0.3 (as those obtained in this study), with an alpha of 0.05 and a power of 80%, a sample size of 84 was needed; thus, our sample size of 92 was appropriate.

## 5. Conclusions

In conclusion, these results agree with those of previous reports of increased PLMS and add new knowledge on the prevalence of LLM and RSD in children born prematurely. These findings suggest that prematurity may continue to placing some children at risk of persistent sleep-related movement disorders. Sleep fragmentation caused by movement disorders during sleep, such as RSD and PLM, can contribute to daytime sleepiness and affect their overall health, especially their behavior and cognitive functions. The authors would like to recommend screening children who were born prematurely for sleep disorders early on to help establish an early diagnosis and treatment and to prevent further health sequelae.

## Figures and Tables

**Table 1:** Age and gestational age at birth of the subjects enrolled in this study.

	N	Age, Years		Gestational Age, Months	
		Median	Interquartile Range	Median	Interquartile Range
Total	92	4.0	2.0–6.0	31.0	27.0–34.0
F	30	4.5	2.0–8.0	31.5	27.0–34.0
M	62	3.0	2.0–6.0	30.5	26.0–34.0

**Table 2:** Descriptive statistics of PSG parameters in children born prematurely.

	Median	Minimum	Maximum	Lower Quartile	Upper Quartile
Total sleep time, min	453.5	182.0	579.0	423.0	493.5
Sleep latency, min	25.5	0.0	159.0	11.0	45.0
Sleep efficiency, %	87.0	41.0	98.0	82.0	92.0
Sleep stage N1, %	7.0	0.9	29.0	3.0	11.0
Sleep stage N2, %	40.5	14.0	70.0	33.0	48.0
Sleep stage N3, %	31.0	14.0	57.0	25.0	39.5
Sleep stage R, %	19.0	0.0	29.0	14.5	23.0
Arousal index, n/h	11.0	3.9	35.0	8.0	13.5
Obstructive AHI, n/h	1.6	0.0	21.0	0.7	3.7
Central AHI, n/h	0.9	0.0	6.5	0.3	1.9
PLMS index, n/h	1.4	0.0	30.7	0.0	3.3
LMM index, n/h	3.6	0.0	14.5	1.7	6.0

PLMS = periodic leg movements during sleep; AHI = apnea/hypopnea index; LMM = large muscle group movements.

**Table 3:** Results of the multiple regression analysis between PLMS index or LMM index (dependent variables) and selected PSG parameters (independent factors) in children born prematurely.

PLMS Index				LMM Index			
	Correlation	R^2^	*p*		Correlation	R^2^	*p*
Age	0.026	0.182	0.902	Age	0.254	0.127	0.221
GA at birth	0.192	0.040	0.357	GA at birth	−0.067	0.072	0.749
Sleep efficiency	−0.217	0.345	0.297	Sleep efficiency	0.019	0.376	0.928
Arousal index	0.054	0.391	0.798	Arousal index	0.321	0.323	0.118
oAHI	−0.404	0.280	0.045	oAHI	−0.397	0.284	0.049
LMM index	−0.143	0.250	0.495	PLMS index	−0.143	0.217	0.495

GA = gestational age; oAHI = obstructive apnea/hypopnea index; LMM = large muscle group movements; PLMS = periodic leg movements during sleep.

## Data Availability

The data presented in this study are available on request from the corresponding author. They are not publicly available due to restrictions, e.g., privacy or ethical.
